# Serum ionized calcium: evaluation of the Analyte +2 calcium analyser in a clinical chemistry laboratory

**DOI:** 10.1155/S146392468600038X

**Published:** 1986

**Authors:** Rosemarie Freaney, Orla M. Casey, Teresa Quinn, Francis P. Muldowney

**Affiliations:** Department of Metabolism & Renal Disease St. Vincent's Hospital & Department of Medicine University College Dublin Ireland


Journal of Automatic Chemsitry/*Journal of Clinical Laboratory Automation*, Vol. 8, No. 4 (October–December 1986), pp. 197–201

### Serum ionized calcium: evaluation of the Analyte +2 calcium analyser in a clinical chemistry laboratory

**Rosemarie Freaney, Orla M. Casey, Teresa Quinn and Francis P. Muldowney**

*Department of Metabolism & Renal Disease, St. Vincent's Hospital & Department of Medicine, University College, Dublin, Eire*

### Introduction

Calcium is present in serum in three forms—protein bound, complexed and ionized or free. The ionized fraction is the metabolically active one and many important physiological processes, such as muscle contraction, blood coagulation, membrane permeability and parathyroid hormone secretion, are known to depend on calcium ion activity (or concentration) in serum.

Determination of serum ionized calcium (Cai) is very helpful in the diagnosis of parathyroid disorders [1 and 2]. Since the measurement of calcium ion activity is theoretically independent of albumin concentration, it is particularly useful in assessing calcium status in clinical conditions associated with gross change in serum proteins, i.e. multiple myeloma, protein losing intestinal or renal disease and liver disease. Rapid infusion of citrated blood has been shown [3] to cause acute lowering of serum ionized calcium. Monitoring of this parameter is therefore important in transplant and other major surgical procedures.

The development of an electrode capable of determining calcium ion activity in the presence of excess potassium, sodium and ammonium was first reported by Ross [4] in 1967. Initial measurements were made with a static or dip-type electrode. Anaerobic measurements became possible with Orion flow-through electrodes in 1970 [5], and since that time a number of instruments/analysers capable of measuring ionized calcium have been produced.

The present communication reports an evaluation of the recently developed Analyte +2 system, which provides simultaneous pH and calcium ion activity measurements. It also compares these results with values obtained using the Orion SS 20 calcium analyser.

### Materials and methods

### *Instrument description*

The Analyte +2 System (Baker Instruments) is a flow-through, ion selective, electrode-based analyser designed for *in vitro* diagnostic testing, and capable of performing ionized calcium and pH measurements on undiluted serum, plasma and whole blood and also capable of displaying ionized calcium values corrected to pH 7·40 [6]. Measuring time from sample injection to read-out is 75 s.

Sample and reagent are pumped through the system by means of a peristaltic pump which cycles the fluid through the electrode stack, and finally out to waste.

The calcium, pH and reference electrodes are separate entities located within the electrode assembly.

The calcium electrode consists of a plasticized vinyl membrane with an organo-phosphorous salt used as an ion exchange 'ionophore'.

The pH electrode is a pH-sensitive glass membrane, and the reference electrode is silver/silver chloride, with an internal solution of 1·5 M potassium chloride providing a fixed ion concentration.

Results are displayed and printed as ionized calcium, corrected ionized calcium, and pH values.

The system can be operated in two modes by means of an autocalibration switch: the standby/ready mode means that calibration can only be obtained on demand; in the calibration/stat mode an automated two-point calibration is performed if the analyser is idle for 20 consecutive minutes.

The recommended daily maintenance requires only 5 min of operator time. An aid in trouble-shooting is the facility to obtain a print-out of the electrode potentials. All parts of the Analyte +2 which require replacement are readily accessible, so replacement of tubing, selection valve seals or electrodes, is a simple procedure.

### *Sample preparation*

*Serum:* blood was drawn into vacutainer tubes (no anticoagulant). Following centrifugation, the stopper was removed from the vacutainer and the serum quickly drawn into a 2 ml plastic syringe. The syringe needle was then embedded in a rubber bung and stored at 4 °C to await analysis.

*Whole blood:* venous blood was drawn into a vacutainer containing a dilution of Calcium-Heparin S 4500 (Radiometer A/S Copenhagen), chosen to give a final concentration of 9 I.U. per ml of whole blood. This heparinized whole blood was then drawn into a 2 ml syringe and analysed immediately.

### Statistical analysis

The statistical procedure for precision assessment was by calculation of means and standard deviations of replicate values. Linear regression analysis was done by the method of least squares.

### Results

### *Analytical precision*

Precision was assessed by replicate analysis of human serum, horse serum and commercial reference materials —Monitrol, Monipath, Baker Normal and Abnormal chemistry controls. For the assessment of within-day precision, ionized calcium was measured 30 times for each control. Precision was also assessed for aqueous calcium solutions ($N = 30$). Between-day precision was calculated from the results of five analyses of both aqueous and serum-based samples each day for 20 days. The results (tables 1 and 2) show that within batch co-efficient of variation (CV) for three of four aqueous calcium solutions measured was below 0·5% and for the remaining one was 1·28%. The between-batch CV tended to be greater, illustrating the day-to-day variability in electrode response to both aqueous and serum-based materials. Precision of the serum-based controls varied with the materials used. The results in table 2 indicate good precision for three reference materials (CV 1·1–1·8%). Significant variation occurred with one control, which gave a between-batch CV of 6·0% for the normal chemistry control and 11·2% for the abnormal control. A high degree of precision is evident (tables 1 and 2) for pH determination in all reference materials. Where imprecision was apparent in ionized calcium measurement, this remained despite correction of ionized calcium value to pH 7·40.

*Table 1. Within-day precision for ionized calcium.*

| | Ionized calcium | | | | Ionized calcium corrected | | | | pH | | | |
|---|---|---|---|---|---|---|---|---|---|---|---|---|
| Reference material | *N* | Mean | SD | CV % | *N* | Mean | SD | CV % | *N* | Mean | SD | CV % |
| Aqueous | | | | | | | | | | | | |
| Baker low standard | 30 | 1·204 | 0·005 | 0·41 | 30 | 1·224 | 0·005 | 0·40 | 30 | 7·45 | 0 | 0 |
| Baker high standard | 30 | 2·43 | 0·007 | 0·30 | 30 | 2·206 | 0·010 | 0·47 | 30 | 7·097 | 0·005 | 0·08 |
| Calcium standard (Orion) | | | | | | | | | | | | |
| Low | 30 | 0·52 | 0·007 | 1·28 | 30 | 0·48 | 0·006 | 1·27 | 30 | 7·138 | 0·008 | 0·12 |
| High | 30 | 1·98 | 0·006 | 0·32 | 30 | 1·77 | 0·006 | 0·31 | 30 | 7·059 | 0·007 | 0·10 |
| Serum based | | | | | | | | | | | | |
| Monitrol | 30 | 1·31 | 0·010 | 0·73 | | | | | 30 | 6·70 | 0·006 | 0·08 |
| Baker abnormal chemistry control | 30 | 0·506 | 0·037 | 7·25 | 30 | 0·686 | 0·053 | 7·76 | 30 | 8·31 | 0·019 | 0·23 |
| Baker normal chemistry control | 30 | 0·82 | 0·006 | 0·71 | 30 | 1·09 | 0·008 | 0·72 | 30 | 8·25 | 0·008 | 0·10 |
| Human serum | 29 | 1·18 | 0·012 | 1·01 | 29 | 1·20 | 0·007 | 0·60 | 29 | 7·46 | 0·02 | 0·26 |
| Horse serum (BW) | 30 | 1·04 | 0·009 | 0·83 | 30 | 1·07 | 0·010 | 0·98 | 30 | 7·50 | 0·031 | 0·01 |
| Moni path | 30 | 1·69 | 0·008 | 0·47 | | | | | 30 | 6·96 | 0·003 | 0·40 |

*Table 2. Between-day precision for ionized calcium.*

| | Ionized calcium | | | | Ionized calcium corrected | | | | pH | | | |
|---|---|---|---|---|---|---|---|---|---|---|---|---|
| Reference material | *N* | Mean | SD | CV % | *N* | Mean | SD | CV % | *N* | Mean | SD | CV % |
| Aqueous | | | | | | | | | | | | |
| Baker low standard | 101 | 1·202 | 0·006 | 0·52 | 101 | 1·222 | 0·006 | 0·51 | 101 | 7·450 | 0 | 0 |
| Baker high standard | 100 | 2·400 | 0·018 | 0·74 | 100 | 2·171 | 0·018 | 0·84 | 100 | 7·089 | 0·013 | 0·18 |
| Calcium standard (Orion) | | | | | | | | | | | | |
| Low | 100 | 0·515 | 0·018 | 3·45 | 100 | 0·470 | 0·017 | 3·65 | 100 | 7·112 | 0·015 | 0·21 |
| High | 90 | 1·999 | 0·015 | 0·75 | 22 | 1·789 | 0·024 | 1·37 | 90 | 6·847 | 0·156 | 2·27 |
| Serum based | | | | | | | | | | | | |
| Monitrol | 100 | 1·298 | 0·021 | 1·65 | — | — | — | — | 100 | 6·698 | 0·026 | 0·39 |
| Baker abnormal chemistry control | 100 | 0·620 | 0·069 | 11·16 | 100 | 0·799 | 0·074 | 9·28 | 100 | 8·196 | 0·064 | 0·78 |
| Baker normal chemistry control | 100 | 0·878 | 0·052 | 5·97 | 100 | 1·118 | 0·046 | 4·13 | 100 | 8·154 | 0·067 | 0·82 |
| Moni path | 100 | 1·698 | 0·019 | 1·11 | 100 | — | — | — | 100 | 6·965 | 0·031 | 0·45 |
| Horse serum | 60 | 1·054 | 0·019 | 1·80 | 60 | 1·088 | 0·009 | 0·82 | 60 | 7·497 | 0·067 | 0·90 |

*Selectivity of the calcium electrode*

The effect on altering sodium, potassium and magnesium within the physiological range was assessed by addition of weighed amounts of each to a buffered aqueous calcium standard containing 1·2 mmol/l calcium and 120 mmol/l sodium. Potassium and magnesium caused negligible change in ionized calcium values, and the small decrease in measured Cai produced by the addition of sodium is shown in figure 1. Ionized calcium decreased by 0·008 mmol/l per 10 mmol/l increment in sodium concentration.

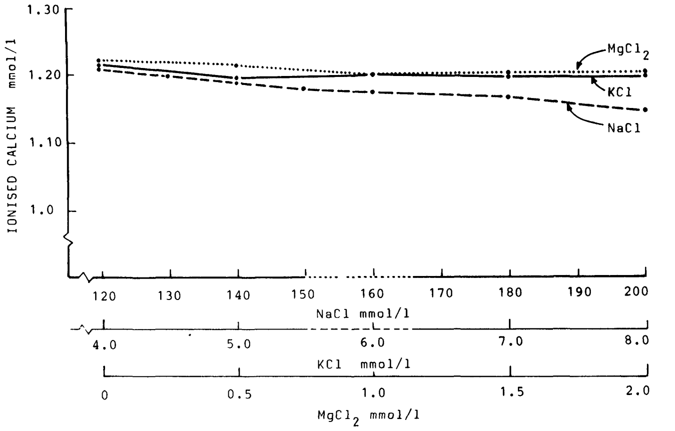

*Figure 1. Effect of alterations in sodium, potassium and magnesium concentration on measured ionized calcium.*

### Accuracy

Accuracy is difficult to assess as there is no absolute reference method available and also because calcium added to serum for recovery experiments binds to protein, and also forms complexes. 'Accuracy' in this study was defined by two methods: (1) Comparison of Analyte +2 ionized calcium values with the values quoted for commercial reference sera (see table 3). (2) Comparison of Analyte +2 calcium values for reference materials and patient sera with values obtained using another calcium selective electrode—the Orion SS 20. Table 3 shows the measured values and expected ionized calcium values (where quoted by the manufacturer) for reference materials on both analysers.

Ionized calcium in 106 sera was determined on both the Analyte +2 and the Orion SS20 calcium analysers. The data (figure 2) was analysed by linear regression and least squares method, and the correlation co-efficient was $r = 0{\cdot}986$, ($P < 0{\cdot}001$). The regression equation was $y = 0{\cdot}027 + 1{\cdot}02x$ where $y$ represents the Analyte +2 values. Calculation of the slope by the Deming method of linear regression [7], yielded a value of 1·035, compared to a regression coefficient of 1·02 for the least squares method.

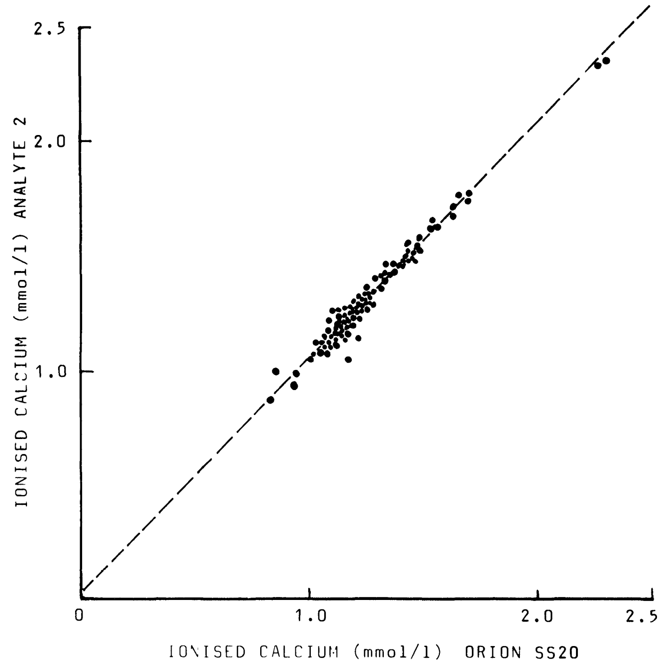

*Figure 2. Correlation of ionized calcium concentration determined by Orion SS 20 Calcium analyser (x) and Analyte +2 (y) in 106 human sera. Correlation co-efficient was $r = 0{\cdot}986$ and regression equation $y = 0{\cdot}027 + 1{\cdot}02x$. 95% confidence limits = 1·020 +/− 0·034 mmol/l.*

*Table 3. Ionized results from Analyte 2 and Orion SS20 analysers (Mean +/− SD).*

| Reference material | N | Analyte 2 measured value (mmol/l) | | N | Orion SS 20 measured value (mmol/l) | | Manufacturers' quoted value (mmol/l) |
|---|---|---|---|---|---|---|---|
| Monitrol (202) | 18 | 1.42 | 0·02 | 18 | 1·41 | 0·02 | 1·29–1·45[1] |
| Monitrol (207) | 55 | 1·30 | 0·02 | 5 | 1·35 | 0·04 | 1·18–1·32[1] |
| Monipath | 6 | 1·73 | 0·03 | 6 | 1·71 | 0·03 | No quoted value |
| Baker normal chemistry | | | | | | | |
| Ionized Ca | 30 | 0·85 | 0·04 | 6 | 1·03 | 0·03 | 0·81–0·89[2] |
| 'Corrected' Ca | | 1·09 | 0·04 | | — | | 1·06–1·12[2] |
| Baker abnormal | | | | | | | |
| Ionized Ca | 49 | 0·62 | 0·09 | 14 | 0·71 | 0·07 | 0·53–0·81[2] |
| 'Corrected' Ca | | 0·80 | 0·10 | | — | | 0·70–0·98[2] |

(1) Manufacturer's value obtained on Nova II analyser—number of determinations not stated.
(2) Analyte 2 system—values based on not less than 30 samples.

### *Validation of clinical results*

Serum ionized calcium values for patients classified as hypercalcaemic, normocalcaemic and hypocalcaemic, according to their serum total calcium levels, are shown in figure 3. All hypercalcaemic patients also had increased ionized calcium values and hypocalcaemic patients had reduced Cai levels as would be expected when serum albumin levels are normal. Total calcium concentration, however, is critically dependent on the concomitant level of serum protein. Moore [5] reported that approximately 39·5% of total calcium is protein bound (mainly to serum albumin). Therefore, the measurement of serum Cai, which is theoretically independent of, or minimally affected by, albumin concentration should be used to assess calcium status where abnormalities in serum proteins are expected. Significant decrease in serum protein can occur in patients with protein losing renal and intestinal disease and in liver disease, while multiple myeloma and prolonged venostasis are associated with increased serum protein levels. A number of algorithms that correct total calcium for albumin concentration have been proposed. Ladenson [1] tested the ability of several correction factors for total calcium to predict Cai levels and concluded that no single correction factor was useful in all clinical conditions—he recommended the determination of serum Cai to assess calcium status.

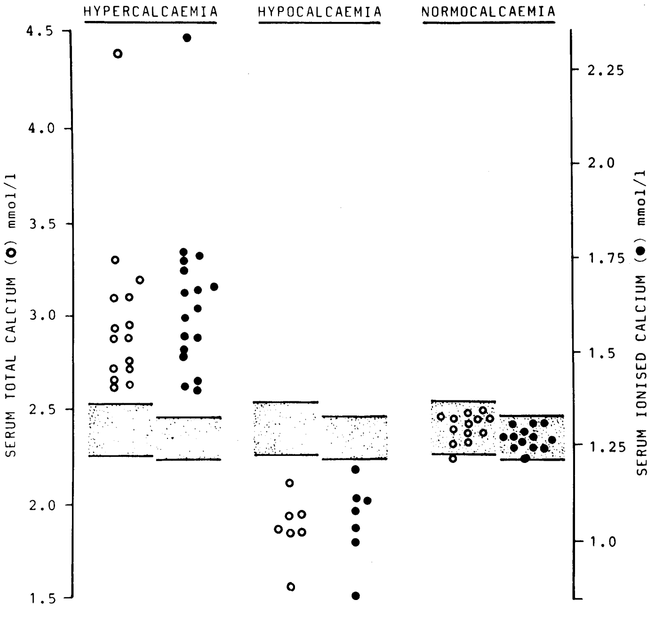

*Figure 3. Ionized serum calcium (●) (Analyte +2) and total serum calcium (○) in hypercalcaemic, hypocalcaemic and normocalcaemic subjects.*

### *Stability of ionized calcium*

Measurements of pH and ionized calcium were made in serum samples at time 0 and after 2, 4 and 24 h at 4 °C. Table 4 shows the mean change (△) +/−SD from zero time in each case. Stability during longer-term storage (11 days) was also documented. Table 4 shows that samples could be stored at 4 °C for two days without clinically significant change (−0·005 +/−0·010 mmol/l) in serum ionized calcium. Serum pH, however, was less stable and a mean change of 0·014 +/−0·016 units occurred after 24 h.

### *Reference range*

Serum ionized calcium was assayed in 29 healthy controls (M12 F17, age range 20–30 years). The mean value was 1·26 +/− 0·034, range 1·19 − 1·33 +/− 2 SD.

### *Comparison of ionized calcium values in serum and whole blood*

Ionized calcium was measured simultaneously in whole blood and serum ($N = 23$) and close correlation between samples was obtained. The correlation co-efficient was $r = 0{\cdot}997$ and regression equation was $y = 1{\cdot}02x - 0{\cdot}05$, where $y$ = serum ionized calcium.

### Discussion

Alteration of serum ionized calcium may be caused by a disturbance of acid base status [5], or by a disorder of calcium metabolism [1]. If an *in vitro* pH change occurs in serum before or during analysis, the ionized calcium value recorded may not reflect the actual level in the patient. Methodology and instrumentation have been developed along two distinct lines to deal with this problem. In the first approach, the serum sample is equilibrated with $CO_2$ gas to produce a standard pH (usually 7·40) at which ionized calcium is measured. By mathematical manipulation the ionized calcium values may be corrected to give the patient's ionized calcium level at his original pH, if this is known [5]. In the second approach, precautions are taken to prevent $CO_2$ loss and consequent pH change during both sample handling and storage [1]. The ionized calcium value then measured should reflect the actual ionized calcium status of the patient.

The Analyte +2 adopts the latter approach and indicates the patient's ionized calcium levels at his prevailing pH. In addition, it displays the sample pH and corrects the Cai value already measured to the ionized calcium value which would occur at a serum pH of 7·40.

*Table 4. Effect of storage on serum ionized calcium and pH.*

| Storage conditions | | n | 2 h | n | 4 h | n | 24 h | n | 2 days | n | 4 days | n | 7 days | n | 8 days | n | 11 days |
|---|---|---|---|---|---|---|---|---|---|---|---|---|---|---|---|---|---|
| Stored in syringe at 4 °C | △ Serum Cai | 19 | −0·009 +/−0·010 | 17 | −0·011 +/−0·015 | 27 | 0·000 +/−0·011 | 6 | −0·005 +/−0·010 | 7 | −0·010 +/−0·012 | 11 | −0·005 +/−0·016 | 10 | +0·008 +/−0·017 | 9 | −0·021 +/−0·030 |
| | △ Serum pH | 19 | +0·002 +/−0·006 | 17 | +0·004 +/−0·009 | 17 | +0·014 +/−0·016 | 6 | +0·023 +/−0·016 | 7 | +0·021 +/−0·011 | 11 | +0·037 +/−0·021 | 9 | +0·037 +/−0·021 | 9 | +0·040 +/−0·027 |

Mean change (△) observed from results of analyses performed immediately on separation of serum (0 time).

This report provides data on pH and Cai change during storage of serum, in addition to the evaluation of precision and accuracy of the assay technique.

The stability studies performed showed that serum could be stored with minimal change in pH for 4 h. After two days' storage, despite pH increase, 95% of Cai values would still lie within 0·025 mmol/l (mean +/− 2SD) of their original values, and would yield useful clinical information. Schwartz [6], Moore [5], Pederson [8] and Wybenga [9] reported an approximate decrease of 0·05 mmol/l in Cai per 0·1 unit pH increase. Using this figure, the theoretical change in Cai after two days would be 0·012 mmol/l. The actual measured decrease in Cai was 0·005 +/− 0·010 mmol/l.

Mean serum ionized calcium in healthy subjects in this study was similar to that previously published by Johnson [10] for the Analyte +2 analyser.

The precision of measurements using the Analyte +2 was adequate for aqueous calcium solutions, for human serum, and most serum-based controls. The imprecision recorded in some instances for the Baker reference material does not appear to be related to pH. An additional feature was the variation of within-day precision (CV 0·71%) and between-day precision (CV 5·97%) for the Baker normal chemistry control. This could reflect vial-to-vial variability.

In most cases the ionized calcium values obtained for the reference materials used fell within the manufacturers' quoted ranges. However, these ranges were wide and did not always state the number of results from which they were established. These ranges are consensus values, rather than absolute values for ionized calcium activity or concentration in serum.

A recent publication [11] comparing Cai values obtained by five different calcium analysers (not including the Analyte +2) showed good agreement between the Orion SS 20, the Nova 2 and the ICA 1 analyser results. The present report shows close correlation between the Orion SS 20 and the Analyte +2 values.

Ionized calcium measurements are required rapidly during transplant surgery involving massive blood transfusion. For this reason, a comparison of ionized calcium levels in serum and whole blood was made. The close correlation reported here permits analysis of whole blood in an emergency. The short interval between injection of sample and read-out of result on the Analyte +2 makes this analyser especially useful during transplant surgery.

Reagent consumption was examined both in the calibration/stat and standby/ready modes during the period of instrument assessment. Reagent cost was high (approximately £90 per week) and this could be reduced (to approximately £65) when the Analyte +2 was used in the standby/ready mode. This was based on a sample throughput of 375 samples per week.

It was not possible to experimentally verify electrode life during the period of instrument assessment. The manufacturer claims a minimum lifespan of six months for the calcium electrode and nine to twelve months for pH and reference electrodes. An accurate costing exercise would require a period of 18 months to two years to complete. However, electrodes have been in operation for 10 months and show no significant deterioration in electrode parameters as displayed on the instrument print-out. Replacement of electrodes would cost approximately £400 for the calcium electrode, with pH and reference electrodes costing approximately £350 and £250 respectively. The Analyte +2 running costs compare favourably with the cost for the Orion SS 20, being one third less over the short period studied.

Ionized calcium is therefore not an inexpensive test. However, in many clinical conditions, it is the method of choice to assess calcium status, and during liver transplantation surgery it is a vital requirement.

In summary, the Analyte +2 system is easy to use, provides rapid monitoring of serum Cai and pH and requires little routine maintenance.

### References

1. Ladenson, J. H. and Bowers, G. N., *Clinical Chemistry*, **19** (1973), 575.
2. Muldowney, F. P., Freaney, R., Spillane, E. A. and O'Donohoe, P., *Irish Journal of Medical Science*, **142** (1973), 223.
3. Hinkle, J. E. and Cooperman, L. H., *British Journal of Anaesthesia*, **43** (1971), 1108.
4. Ross, J. W., *Science*, **156** (1967), 1378.
5. Moore, E. W., *Journal of Clinical Investigation*, **49** (1970), 318.
6. Schwartz, H. D., McConville, B. C. and Christopherson, E. F., *Clinica Chimica Acta*, **31** (1971), 97.
7. Payne, R. B., *Clinical Chemistry*, **30** (1984), 807.
8. Pederson, K. O., *Scandinavian Journal of Clinical Laboratory Investigation*, **25** (1970), 223.
9. Wybenga, D. R., Ibbott, F. A. and Cannon, D. C., *Clinical Chemistry*, **22** (1976), 1009.
10. Johnson, L., Graham, G. and Brueningsen, D. A., *American Clinical Products Review*, July (1985), 24.
11. Uldall, A., FoghAndersen, N., Thode, J., Boink, A. B. T. J. and Kofstad, J. *et al.*, *Scandinavian Journal of Clinical Laboratory Investigation*, **45** (1985), 255.

